# VT-1161—A Tetrazole for Management of Mono- and Dual-Species Biofilms

**DOI:** 10.3390/microorganisms11020237

**Published:** 2023-01-17

**Authors:** Angela Maione, Aldo Mileo, Stefano Pugliese, Antonietta Siciliano, Luigi Cirillo, Federica Carraturo, Elisabetta de Alteriis, Maria De Falco, Marco Guida, Emilia Galdiero

**Affiliations:** 1 Department of Biology, University of Naples “Federico II”, Via Cinthia, 80126 Naples, Italy; 2 Department of Neurosciences, Reproductive Sciences and Odontostomatology, University of Naples “Federico II”, 80126 Naples, Italy; 3 National Institute of Biostructures and Biosystems (INBB), 00136 Rome, Italy; 4 Center for Studies on Bioinspired Agro-Environmental Technology (BAT Center), 80055 Portici, Italy

**Keywords:** antifungal drugs, polymicrobial biofilms, *Klebsiella pneumoniae*, *Staphylococcus aureus*, *Candida albicans*, antimicrobial resistance, *Caenorhabditis elegans*

## Abstract

VT-1161 is a novel tetrazole antifungal agent with high specificity for fungal CYP51 (compared to human CYP enzymes) which has been proven to have fewer adverse effects and drug–drug interaction profiles due to fewer off-target inhibitors. In this study, we evaluated the anti-biofilm potential of VT-1161 against mono- and dual-species biofilms of *Candida albicans*, *Klebsiella pneumoniae* and *Staphylococcus aureus*. VT-1161 inhibited planktonic growth of all three strains, with an MIC value of 2 µg mL^−1^ for *C. albicans* and 0.5 µg mL^−1^ for *K. pneumoniae* and *S. aureus*, and killed 99.9% of the microbial populations, indicating a cytocidal action. Additionally, VT-1161 showed an excellent anti-biofilm action, since it inhibited mono-microbial biofilms by 80% at 0.5 µg mL^−1^, and dual-species biofilms of *C. albicans/K. pneumoniae* and *C. albicans/S. aureus* by 90% at the same concentration. Additionally, the eradication of mature biofilms after 24 h of VT-1161 exposure was excellent, reaching 90% at 2 μg mL^−1^ for both mono- and dual-species biofilms. In such mixed biofilms, the use of VT-1161 was revealed to be an alternative treatment because it was able to reduce the number of cells of each species during both inhibition and eradication. Since long-term therapy is necessary for most fungal biofilm infections due to their recurrence and obstinacy, VT-1161 showed low cytotoxicity against normal human cell lines and also against the invertebrate model *Caenorhabditis elegans*. Considering the excellent anti-biofilm potential and its GRAS (generally recognized as safe) status, VT-1161 may find use in the prevention or therapeutic treatment of mono- or poly-microbial biofilms.

## 1. Introduction

Biofilms are characterized by a population of microorganisms that switch from planktonic to sessile state after adhering to different surfaces. Biofilms are so well organized as to allow microorganisms to survive in hostile conditions, providing resistance to the host’s defense and antimicrobial agents. Moreover, once formed, they are difficult to remove leading to life-threatening and recurrent infections in patients [1].

*C. albicans* is the predominant fungal species associated with biofilm formation, and several chronic candida-related diseases are associated with a well-structured biofilm development that can be established on both biotic and abiotic surfaces. As a result, the high degree of the resistance associated with the biofilm interferes with the effectiveness of available therapies [2].

Although microorganisms can form single-species biofilms, it is much more common to find two or more bacterial and/or fungal species in a biofilm. These polymicrobial biofilms often provide specific advantages to each species when compared with single-species biofilms, playing an important role in antimicrobial resistance. *C. albicans* coexists with many different bacterial species [3], and its interactions with the Gram-negative *Klebsiella pneumoniae* and Gram-positive *Staphylococcus aureus* are also discussed in several searches because they exhibit severe antimicrobial resistance and have been associated with mortality and morbidity [4].

For example, an increase in virulence has been observed in *C. albicans–S. aureus* dual-species interactions, with a higher host mortality compared with infections with each species alone [5]. *C. albicans* and *K. pneumoniae* are often isolated together, especially in patients with oral infections or respiratory diseases [6]. Our previous results demonstrated that in the mixed biofilm *C. albicans*/*K. pneumoniae*, there was an interconnection between the two microorganisms, leading to the formation of a strong sessile community wherein the fungus was initially (after 24 h) dominant, but the two species were equally present in the biofilm at 48 h [7]. The strict interactions existing between the two microorganisms were also shown by metabolomics data of the *C. albicans*/*K. pneumoniae* biofilm [8]. Indeed, in the early phase of the dual-species biofilm, the prevalence of *C. albicans* was confirmed by an active glucose metabolism with an upregulation of arabitol and a downregulation of leucine, probably due to an inhibition of the specific *K. pneumoniae* transport system of this amino acid. Instead, in the mature biofilm’s development, an increase of stress-related metabolites (vitamin B6 and trehalose) was observed, explaining the relative reduction in the *C. albicans/K. pneumoniae* ratio during biofilm maturation.

Azoles (e.g., fluconazole) inhibit ergosterol biosynthesis, causing the production of toxic intermediates of the sterol pathway. They are fungistatic and are among the most common antifungals used to treat both systemic and topical infections. Repeated use of fluconazole to treat recurrences increases the risk of resistant isolates’ development. It is now estimated that up to 6% of *C. albicans* isolates are resistant [9].

*C. albicans* biofilms are resistant to the majority of known antifungal drugs, making these infections particularly difficult to combat. The azoles are not effective against *C. albicans* biofilms, and their resistance is multifactorial due to three major factors: the upregulation of efflux pumps, the presence of the extracellular matrix, and the existence of recalcitrant and metabolically inactive “persister” cells [10].

Therefore, researchers are looking for new antimicrobial agents that could be effective against a wide range of *Candida* biofilms and show less cytotoxicity.

Tetrazole oteseconazole (VT-1161) represents a new generation of fungal CYP51 inhibitor with high specificity for fungal CYP51 compared to human CYP enzymes. [11,12]. VT-1161 showed potent in vitro activity against *C. albicans* in initial tests and against fluconazole-resistant *C. albicans* from acute and recurrent vulvovaginal candidiasis, with MICs ranging from <0.015 to 2 µg mL^−1^, with an MIC_90_ of < 0.015 µg mL^−1^. VT-1161 has proved to be clinically effective and safe not only in a phase 2 study in women with recurrent vulvovaginal candidiasis (RVVC), but also in a phase 3 study on the treatment of culture-verified vulvovaginal candidiasis (VVC) episodes; thus leading to its approval by the FDA in April 2022 [13] (clinicaltrials.gov identifier. NCT03840616, NCT03562156, NCT03561701).

Based on the promising effects described above, the aim of this study was to evaluate the possible anti-biofilm effects of VT-1161 on a five in vitro biofilm model including a single biofilm of *C. albicans*, a single biofilm of *S. aureus*, a single biofilm of *K. pneumoniae* and a dual-species biofilm of *C. albicans*/*S. aureus* and *C. albicans*/*K. pneumoniae*, to evaluate its cytotoxic effects on human cells and in vivo toxicity, and to evaluate its treatment using *Caenorhabditis elegans*. To our knowledge, the anti-biofilm efficacy of VT-1161 on single and dual-species biofilms has not been reported thus far, so this study could contribute to enlarging the potentiality of this drug in antifungal therapy.

## 2. Materials and Methods

### 2.1. Chemicals

VT-1161 was supplied by Target Mol-tebu-bio, Chemicals Inc. (Mont Belvieu, TX, USA) and fluconazole, vancomycin, meropenem were obtained commercially from Sigma-Aldrich Co. (St. Louis, MO, USA). Stock solutions were obtained by dissolving the powders in 5% *v/v* DMSO. at The final concentration was100 µg mL^−1^ for VT-1161, 50 µg mL^−1^ for fluconazole, and 10 µg mL^−1^ for the two antibiotics.

### 2.2. Microorganism Strains and Culture Conditions

Reference strains of three species, including *Candida albicans ATCC 90028, Staphylococcus aureus ATCC 6538, Klebsiella pneumoniae ATCC13883* available in our laboratory were used in this study. Organisms were stored as frozen stocks at −80 °C. *C. albicans* was maintained on YPD plates (Sigma-Aldrich Italia) (1% yeast extract, 2% peptone, 2% glucose and 2% agar), while *S. aureus* and *K. pneumoniae* were maintained on tryptic soy agar plates (TSA, VWR chemicals, Leuven, Belgium). Colonies of the two bacteria were picked up and inoculated into liquid tryptic soy broth (TSB, VWR chemicals, Leuven, Belgium). Colonies of *C. albicans* were picked up and placed in the same medium supplemented with 1% glucose. All the microbial suspensions were stored at 37 °C overnight. Cells were harvested by centrifugation at 5000 rpm at 4 °C for 10 min, followed by washing in phosphate buffered saline (PBS, Oxoid Ltd., Basingstoke, UK) three times. Then, final suspensions were adjusted to 1 × 10^6^ CFU mL^−1^ in TSB supplemented or not with glucose.

#### Cell Cultures

A prostate non tumoral cell line established by immortalization of adult prostate epithelial cells (PNT1A cells, *ECACC 95012614*) and a human prostate carcinoma cell line (LNCaP cells, *ECACC 89110211*) were used in the present study. Both cell lines were cultivated in RPMI medium (R0883; Sigma-Aldrich), supplemented with 10% Fetal bovine Serum (cod. F7524, FBS, Sigma-Aldrich) 2 mM L-glutamine (cod. 59202C; Sigma Aldrich) and 100 U/mL penicillin/streptomycin (cod. P4333; Sigma-Aldrich) in a humidified incubator at 37 °C and 5% CO_2_. After 70% confluency, the cells were enzymatically detached with Trypsin/EDTA solution 0.25% (cod. T4049; Sigma-Aldrich) and cultured into new flasks. The culture medium was replaced twice a week. The cell lines were monitored daily using an inverted microscope.

### 2.3. Antimicrobial Assay (MIC)

The minimum inhibitory concentration (MIC) of VT-1161, vancomycin, meropenem and fluconazole was determined in accordance with a broth microdilution protocol from the Clinical and Laboratory Standards Institute (CLSI M27-A3 and M07-A9) [14,15]. Microbial cultures were incubated at 37 °C for 24 h with one of the following drugs: VT-1161 (range 0.001–2 μg mL^−1^), vancomycin (range 0.1–1 μg mL^−1^), meropenem (range 0.1–1 μg mL^−1^), and fluconazole (range 1–64 μg mL^−1^). Growth was determined at 590 nm wavelength with a microplate reader (SYNERGYH4 BioTek). MICs of drugs were determined as the lowest drug concentration that produced ≥90% inhibition of growth relative to control.

### 2.4. Biofilm Formation and Biofilm Mass Quantification

Biofilms were grown in sterile flat-bottom 96-well microtiter test plates in TSB with or without 1% glucose for 24 h at 37 ◦C, as reported previously [16]. For the dual-species biofilm, the suspensions of the two microorganisms were prepared at a final concentration of 10^6^ cells mL^−1^ (ratio 1:1), dispensed (100 μL per well), and incubated. Single- species or dual-species biofilms were stained with crystal violet (CV) solution as described previously [17]. In brief, biofilms cultured in 96-well plates were air fixed. At total of 0.2% crystal violet solution was then added to each well and incubated for 20 min at room temperature. Excess CV solution was removed by washing with deionized water, and bounded CV was released by 33% *v/v* acetic acid. Optical density was detected at 570 nm (OD_570_) using a microtiter plate reader (SYNERGY H4 BioTek, BioTek Instruments, Inc., Winooski, VT, USA). In accordance with the classification introduced by Stepanović et al., the tested strains were divided into four categories: non-adherent, weakly adherent, moderately adherent and strongly adherent [18]. Each experiment was repeated in triplicate.

### 2.5. Activity of VT-1161 against Mono- and Dual-Species Biofilms

The ability of VT-1161 to prevent mono and poly-microbial biofilm formation and eradicate mature biofilms, was investigated as previously reported [17].

To determine the inhibition of biofilm formation, different concentrations (range 0.05–0.5 μg mL^−1^) were added to wells together with microorganisms, and each plate was incubated for 24 h at 37 °C. Mono and poly-microbial biofilms were assessed using the CV assay as reported before and determination of colony-forming units (CFUs).

For determination of colony-forming units (CFUs) the treated biofilms were scraped from the bottom of the plates, suspended in PBS (1 mL) and homogenized in vortex (90 s). Serial dilutions were prepared in PBS and plated on rose bengal agar plates supplemented with chloramphenicol (for *C. albicans*) and TSA agar plates supplemented with amphotericin B (for *K. pneumoniae* and *S. aureus*). The agar plates were incubated at 37 °C, and the CFUs counted after 24–48 h.

The influence of VT-1161 on biofilms was assessed as follows: 24 h biofilms were treated with different concentrations of the VT-1161 (range 0.25– 2 μg mL^−1^) and the plates were further incubated at 37 °C for another 24 h. The supernatant was removed, and the total cell biomass and cell viability were determined by CV and CFUs assay.

The percentages of inhibition/eradication were calculated as: % biofilm reduction = (OD_570_ control − OD_570_ sample/OD_570_ control) × 100, where OD_570_ control and OD_570_ sample corresponded to the untreated and treated biofilm, respectively.

### 2.6. Cell Cytotoxicity Assay

A cytotoxicity evaluation was carried out according to our previous protocol [19]; PNT1A and LNCaP cells were seeded in 96-well plate at density of 5 × 10^3^ cells per well in 100 μL of growth medium. After 24 h of starvation, which allowed cell cycle synchronization, fresh medium containing different concentrations of VT-1161 (range 0.1–10 μg mL^−1^) was added to each well. Control cells were incubated with vehicle (PBS). The plates were incubated at 37 °C in a humidified incubator with 5% CO_2_ for a period of 24 h. Then, viable cell yield was determined by a colorimetric method using a 3-[4,5-dimethylthiazol-2-yl]-3,5 diphenyl tetrazolium bromide (MTT) assay which correlates cell metabolic activity with formazan crystal concentration. Briefly, 10 µL of MTT solution (5 mg mL^−1^) was added to each well after treatments to reach a final concentration of 0.5 mg mL^−1^. As a result of 4 h of incubation in a humidified incubator at 37 °C and 5% CO_2_, the formazan crystal formed were dissolved with DMSO. The absorbance of formazan crystal concentrations was read at 570 nm in a microplate reader. The cell viability (%) was calculated as (OD_570_ sample − blank/OD_570_ control-blank) × 100, where OD_570_ control and OD_570_ sample were the untreated and treated cells, respectively. The tests were performed in triplicate.

### 2.7. Adhesion to PNT1A Cells

To study the inhibition of *C. albicans*, *S. aureus*, and *K. pneumoniae*’s adhesion to PNT1A cells monolayers by the VT-1161, cells were pre-treated with the concentrations corresponding to 1 × MIC and 0.5 × MIC of compound for 2 h at 37 °C and 5% CO_2_. After, microorganisms’ inoculation (∼6log CFU/well) was added, and the plate was incubated for 1 h at 37 °C and 5% CO_2_. The infected monolayers were rinsed three times with PBS, and cells were recovered and diluted in PBS. The number of viable adherent microorganisms was determined by serial dilution and plating on respective agar plates as before.

### 2.8. Toxicity Assays in the Nematode Model

To better investigate the toxicity of VT-1161, we used *C. elegans* as a model organism [20]. Briefly, L4 synchronized nematodes had been obtained from adult nematodes after bleaching treatment [21]. About 40 worms were washed with M9 buffer (3 g/L KH_2_PO_4_, 6 g/L Na_2_HPO_4_, 5 g/L NaCl, 0.12 g/LMgSO_4_) and placed into each well of 24-well plates containing M9 buffer (500 µL) with VT-1161 (range 0.5 and 1 μg mL^−1^). Plates were then incubated for 3 days at 25 °C without agitation. Four independent experiments were performed in triplicate. Results are expressed as percentages of live worms, counting the dead nematodes under a microscope (40× magnification).

### 2.9. C. elegans Infection Assay

To evaluate the impact of the single- and dual-species biofilms’ production in the survival of *C. elegans*, synchronous nematodes were washed with M9 buffer, transferred, and placed for 24 h in NGM (nematode growth media) agar plates (60 mm diameter) seeded with microbe inoculum. The concentration of the resulting microbial suspensions was estimated using decimal dilution and adjusted to a final concentration of 1 × 10^6^ CFU mL^−1^ per plate. The worms were then washed five times with M9 buffer to remove extracellular microbes, and transferred to wells (n = 20 per well) containing 2 mL of K-medium (2.36 g/L KCl and 3 g/L NaCl) supplemented with 45 μg/mL Kanamycin). As negative control, L4 *C. elegans* worms were placed on agar plates containing lawns of *E. coli* OP50 strain. The bacterial inoculum was prepared as described above, and the concentration was the same (1 × 10^6^ CFU mL^−1^). The plates were incubated at 25 °C, and nematode survival was examined daily at 24 h intervals for the following six days.

### 2.10. Antivirulence Assays in the Nematode Model

To investigate the effects of VT-1161 on the virulence of *C. albicans*, *S. aureus*, *K. pneumoniae* alone or in combination, pre-infected synchronized adult nematodes were washed with M9 buffer and added into each well containing VT-1161 at concentrations of 0.5 μg mL^−1^. Plates were then incubated at 25 °C without shaking, and the nematode survival was checked every day for 5 days.

### 2.11. Statistical Analysis

All experiments were performed in three biological replicates, where each experiment was done in triplicates Values are shown as mean ± standard deviation (SD). The Kaplan–Meier method were using to plot survival curves. The significant difference between the groups was analyzed using one-way ANOVA followed by Tukey’s or Dunnett’s test. Values with *p* < 0.05 were considered statistically different. GraphPad Prism Software (version 8.02 for Windows, GraphPad Software, La Jolla, CA, USA, www.graphpad.com, accessed on 28 November 2022) was used.

## 3. Results

### 3.1. Determination of Minimum Inhibitory Concentration (MIC)

The impact of VT-1161 on the survival of *C. albicans, K. pneumoniae* and *S. aureus* was assessed using a microbroth dilution method according to the guidelines of Clinical and Laboratory Standards Institute, and is listed in Table 1. VT-1161 showed significant antifungal activity against *C. albicans*, and its MIC value was 2 µgmL^−1^. Therefore, the influence of VT-1161 on *S. aureus* and *K. pneumoniae* growth was also inspected as depicted in Table 1, showing a good antimicrobial activity, with an MIC value of 0.5 µgmL^−1^ displaying a potential antibacterial activity [22,23].

For comparison, the susceptibility of *C. albicans* to fluconazole, as well as that of *K. pneumoniae* and *S. aureus* to meropenem and vancomycin, respectively, is reported in Table 1.

### 3.2. Quantitative Biofilm Production

**Figure 1:**
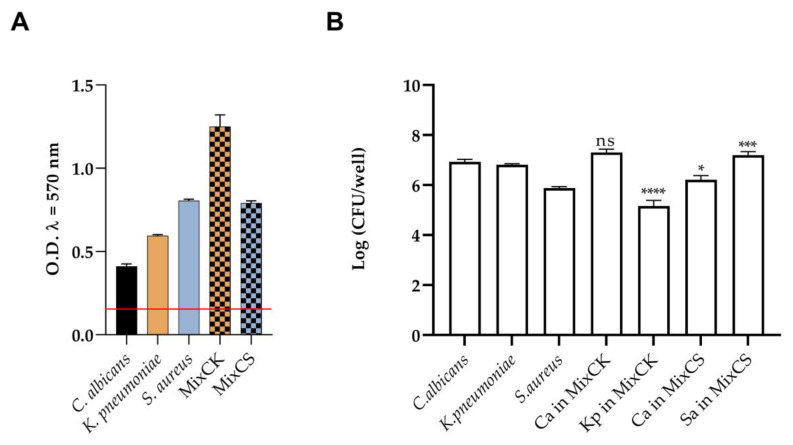
Characterization of mono- and dual-species biofilms of *C. albicans* (Ca), *K. pneumoniae* (Kp) and *S. aureus* (Sa) at 24 h with crystal violet (**A**) and CFUs assay (**B**). The red line represents the ODcut off (mean of negative control + three times the standard deviation). Asterisks indicate difference vs. mono-species biofilm; ns = not significant, * = *p* < 0.05, *** = *p* < 0.001, **** = *p* < 0.0001 (Tukey’s test).

The development of mono- or dual-species biofilms of *C*. *albicans, K. pneumoniae*, and *S. aureus* were confirmed using both CV and CFUs assays. *C. albicans* and the two tested bacteria formed evident biofilms individually as well as when grown together, as shown in Figure 1. The value of ODc (mean of negative control + three times the standard deviation) was 0.1; this was compared with OD_570_ values to classify the microorganisms. *C. albicans* was classified as a moderate biofilm producer (2 × ODc < OD), while *K. pneumoniae* and *S. aureus* were strong biofilm producers (OD ≥ 4 × ODc). Additionally, the two dual species biofilms of *Candida/Klebsiella* (MixCK) and *Candida/Staphylococcus* (MixCS) were robust aggregates (Figure 1A). The vital biomass of mono- and dual-species biofilms is shown in Figure 1B. The growth of *K. pneumoniae* in MixCK biofilm was inhibited by the presence of *C. albicans*; on the contrary, the growth of *S. aureus* in MixCS was stimulated by *C. albicans*.

### 3.3. Inhibition and Eradication of Monomicrobial and Polymicrobial Biofilms

**Figure 2:**
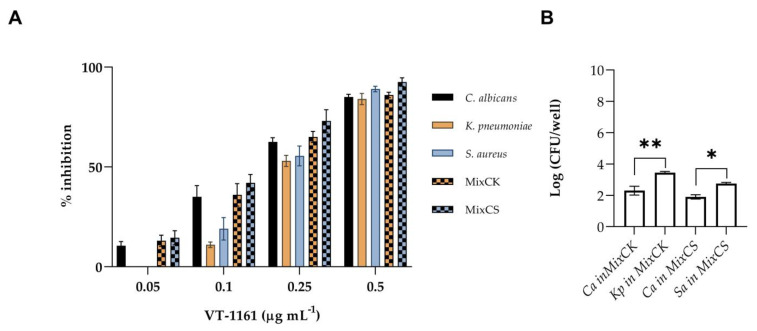
Biofilm inhibition activity of VT-1161 against mono-species biofilm of *C. albicans* (Ca), *K. pneumoniae* (Kp) and *S. aureus* (Sa), and dual-species biofilm of *C. albicans/K. pneumoniae* (MixCK) and *C. albicans/S. aureus* (MixCS); (**A**) % inhibition of total biomass of mono- and dual-species biofilm; (**B**) Log (CFU/well) of mixed biofilm treated with 0.25 µgmL^−1^ of VT-1161. * = *p* < 0.05, ** = *p* < 0.01 (Tukey’s test).

The results of in vitro antibiofilm activity on biofilm formation are shown in Figure 2. The CV assay was used to determine the antibiofilm activity of VT-1161 on monomicrobial and dual-species biofilms. Biofilm formations by *C. albicans*, *S. aureus* and *K. pneumoniae* were significantly inhibited by VT-1161. Both *S. aureus* and *K. pneumoniae* biofilms demonstrated similar patterns of inhibition in concentration-dependent manners. In fact, with the concentration MIC of VT-1161 0.5 µg mL^−1^, biofilm formation was inhibited by 85% or 90%, respectively., VT-1161 was shown to be effective against *C. albicans* even at the sub-MICs concentration of 0.25 µg mL^−1^ inhibiting it by 65%, and reaching an 85% inhibition at concentration of 0.5 µg mL^−1^. Therefore, the results demonstrate a specific prevention activity on *C. albicans* biofilm at sub-MIC concentration, whereas in the case of the bacterial biofilms, the VT-1161 action was due to growth inhibition. We next evaluated the effect of VT-1161 against dual-species biofilms. Mixed species biofilm formation was significantly inhibited by VT-1161 in a concentration-dependent manner (Figure 2A) resulting in inhibition profiles similar to those achieved for monomicrobial biofilms. A total of 70% of dual-species biofilm (MixCK and MixCS) formation was inhibited at concentration of 0.25 µg mL^−1^; however, at a concentration of 0.5 µg mL^−1^ in both cases, an inhibition of biofilm formation of 90% was reached. Given its biofilm-prevention activity, VT-1161 was also tested to determine its antibiofilm activities on mature mono- and dual-species preformed biofilms. With an increase in VT-1161 concentration, a progressive reduction in thepreformed biofilms of the tested species was observed. To determine the composition of the mixed biofilms formed in the presence of VT-1161, the CFUs of the biofilms treated at the concentration of 0.25 µg mL^−1^ were counted. As shown in Figure 2B, the vital biomass of *C. albicans* in the mixed biofilms presented a remarkable reduction in both MixCS and MixCK, even if it was more evident in the MixCS.

**Figure 3:**
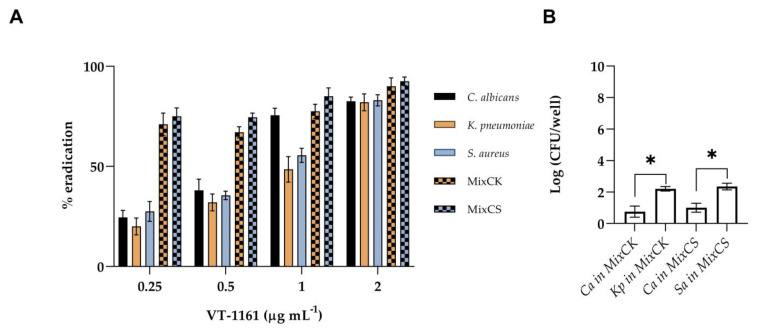
Biofilm eradication activity of VT-1161 against mono-species biofilm of *C. albicans* (Ca), *K. pneumoniae* (Kp) and *S. aureus* (Sa) and dual-species biofilm of *C. albicans/K. pneumoniae* (MixCK) and *C. albicans/S. aureus* (MixCS); (**A**) % eradication of total biomass of mono- and dual-species biofilm; (**B**) Log (CFU/well) of mixed biofilm treated with 0.25 µgmL^−1^ of VT-1161. * = *p* < 0.05 (Tukey’s test).

As showed in Figure 3A, a dose-dependent eradication ability of the VT-1161 on single-species biofilms was observed, reaching 80–90% eradication at the maximum tested concentration of 2 µg mL^−1^. Even if some studies [24] have shown that mature mixed biofilms of bacteria and *Candida* exhibit enhanced resistance compared to single-species biofilms, our result shows a significant reduction in the total biomass of both mixed biofilms, reaching 90% eradication at the highest concentration tested. In Figure 3B, the composition of the mixed biofilms treated with VT-1161 revealed a decrease in *C. albicans* in both mixed biofilms.

### 3.4. Toxicity Assay

**Figure 4:**
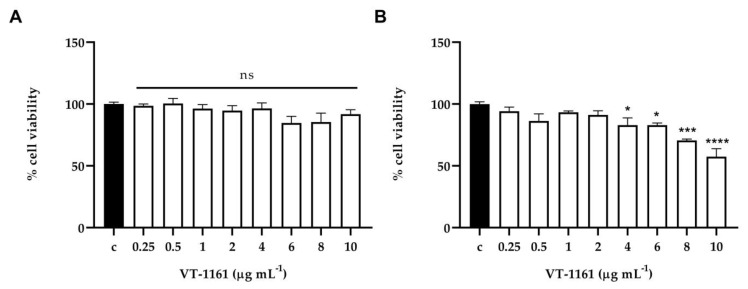
(**A**) MTT assay on the normal cell line (PNT1A) after 24 h of treatment (from 0.25 to 10 µg mL^−1^). No cytotoxicity effect was observed at all concentrations tested compared to the control (PBS). (Dunnet’s test) (**B**) MTT assay on the tumoral cell line (LNCaP) after 24 h of treatment (from 0.25 to 10 µg mL^−1^). Results showed a dose-dependent cytotoxicity effect starting from 4 up to 10 µg mL^−1^ compared to control (PBS). * = *p* < 0,05 *** = *p* < 0.001 **** = *p* < 0.0001 (Dunnet’s test).

**Figure 5:**
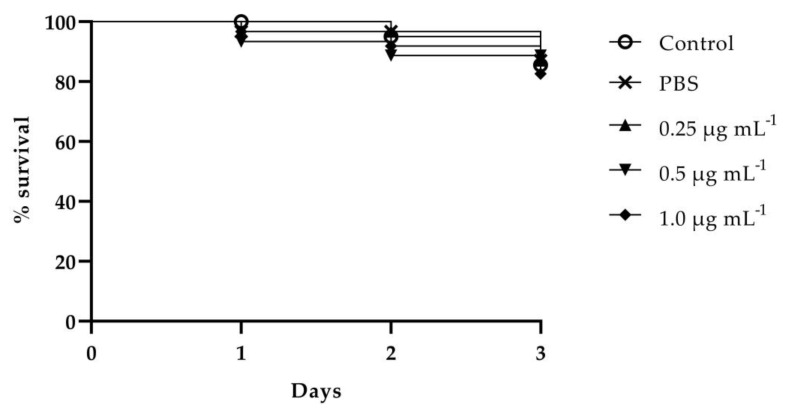
Kaplan–Meier curve showing the survival percentage of *C. elegans* in the presence of VT-1161 (the toxicity of VT-1161 was studied on nematodes, observed using a microscope by determining survival rates for 3 days).

The cytotoxic effect of VT-1161 was determined both on normal human cell lines (PNT1A) and tumoral human cell lines (LNCaP) in vitro and in *C. elegans* in vivo. In vitro results detected by MTT assay showed that the compound did not exhibit significant cytotoxicity on normal cells at all concentrations tested (Figure 4A). On the contrary, VT-1161 displayed a dose-dependent reduction in the cell viability of cancer cells, starting from 4 μg mL ^−1^ up to 10 μg mL ^−1^ and reaching a 43% reduction at the highest concentration tested (Figure 4B). In addition, neither 0.5 nor 1 μg mL ^−1^ VT-1161 displayed a significant effect on the survival of worms after 3-day incubation (Figure 5). These results implied that VT-1161 had low cytotoxicity in vitro and in vivo.

### 3.5. In Vitro and In Vivo Infection Assay

#### 3.5.1. Adhesion Assay on PNT1A Cells

**Figure 6:**
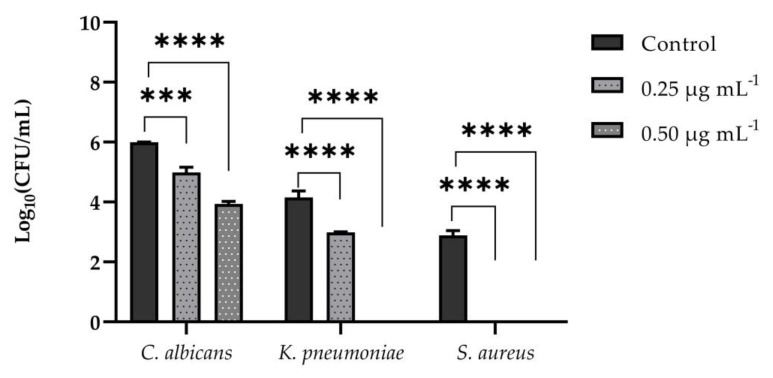
Effect of VT-1161 on the adhesion of *C. albicans*, *S. aureus*, and *K. pneumoniae* to PNT1A cells expressed by CFU mL^−1^. The results were obtained from two independent experiments; *** = *p* < 0.001, **** = *p* < 0.0001 (Tukey’s test).

We also assessed the anti-infective nature of VT-1161 by analyzing *C. albicans, S. aureus*, and *K. pneumoniae* adhesion to PNT1Acells that were pre-treated with it at both concentrations of 0.25 and 0.5 µgmL^−1^. The *C. albicans* counts were reduced from 6 log10 CFU mL^−1^ to 5 log10 CFU mL^−1^ when cells were pre-treated with VT-1161 at a concentration of 0.25 μg mL ^−1^, and from 6 log10 CFU mL^−1^ to 4 log10 CFU mL^−1^ at concentration of 0.5 μg µgmL^−1^. Neither *K. pneumoniae* nor *S.aureus* were detected when cells were pre-treated with a concentration of 0.5 µgmL^−1^ of the compound, while *S. aureus* counts were also undetectable at a concentration of 0.25 µgmL^−1^ (Figure 6). Altogether, these data suggest that VT-1161 could be a potential drug for preventing both fungal and bacterial infections of epithelial cells.

#### 3.5.2. *C. elegans* Infection Assay and Effect of VT-1161 on *C. elegans* Survival

**Figure 7:**
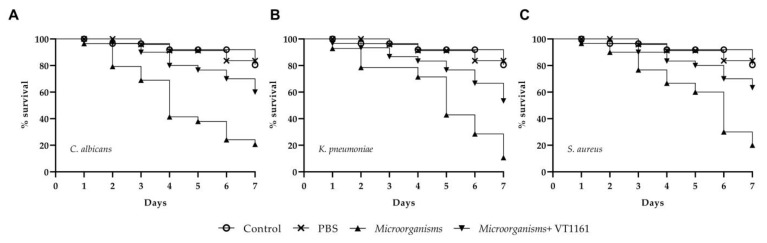
*In vivo* efficacy of VT-1161 at concentration of 0.5 µg mL^−1^ on survival and rescue of *C. elegans* from (**A**) *C. albicans*, (**B**) *S. aureus* and (**C**) *K. pneumoniae* infection.

Firstly, we assessed the ability of three microorganisms to cause infection in *C. elegans* model. The results showed that all species (*C. albicans, S. aureus, K. pneumoniae*) were able to kill *C. elegans* (Figure 7). Lastly, the worms infected were treated with the VT-1161 at a concentration of 0.5x MIC (Figure 7). Exposure to this concentration increased the survival of *C. elegans* in infected worms when compared to the untreated control group. At four days post-infection, 80–85% of the infected and treated larvae survived.

## 4. Discussion

Due to the rapid development of drug-resistant strains, infectious diseases are a major global health concern and new effective antimicrobial agents need to be identified. Bacterial–fungal interaction is accepted and held responsible for relevant human infections. Clinical *C. albicans* isolates continuously increase their tolerance against various commercially used antimycotics, reaching very high MIC values (fluconazole 0.03 to 16 μg mL^−1^; amphotericin-B 0.125 to 4 μg mL^−1^). Notwithstanding, they are considered the golden standard for candidiasis, irrespective of the many side effects associated with them. For example, treatment with fluconazole often leads to headaches, stomach pains, diarrhea, nausea and vomiting, and skin rashes. Azoles used for long therapy in immunocompromised patients lead to hepatotoxicity and hormonal side effects, such as impotence, muscle and nerve disfunction. Amphotericin-B is nephrotoxic at high doses and can cause nausea, vomiting, fever, hypoxia, and other side effects. So, despite the toxicity, treatment costs and resistance of conventional antifungals, novel antifungals and treatment strategies are required. Furthermore, the prominent characteristics of *Candida* biofilms which allow them to grow resistance to traditional antifungals including amphotericin B and fluconazole have underlined the crucial need for treatments that selectively and efficiently inhibit *C. albicans* biofilms. Therefore, due to the emerging development of multidrug-resistant bacteria, drug-repurposing is progressively attracting significant interest as an alternative approach to discover new potential antibiotics [25,26].

Previous research has demonstrated that VT-1161 was found to exhibit favorable antifungal activity against systemic *Candida* infections, with high potency against the candidal CYP51 target enzyme and minimal interaction with human CYP51 [27]. Studies have also pointed out that VT-1161 has a heme interaction and induces a reduction in ergosterol content, thus inhibiting the sterol biosynthesis pathway at a concentration of only 0.004 µg ml^−1^, while not inhibiting human CYP51 activity until a concentration of 263.5 µg ml^−1^ [28,29,30]. Moreover, it is also reported that the reduction in ergosterol content was greater than that caused by an equal concentration of voriconazole, an expected attribute for any new anti-fungal drug candidate, together with the very high selectivity observed [12].

We report the ability of VT-1161 to inhibit and to eradicate fungal biofilms. According to our knowledge, this is the first study investigating VT-1161 activity against single species biofilms of *C. albicans*, but particularly *C. albicans* gram-negative and *C. albicans* gram-positive bacteria multispecies biofilms. In particular, in our study, all strains were susceptible to VT-1161, showing an MIC of 0.5 μg mL ^−1^ for bacteria and an MIC of 2 μg mL ^−1^ for *C. albicans*; *C. albicans* susceptibility to VT-1161 was shown to be slightly higher than those obtained in the global surveillance studies. The use of drugs with different structures from those applied in the clinical treatments of bacterial infections is a well-known potential solution to the antibiotic resistance crisis. It was reported previously that some azoles including imidazole, pyrazole, oxazole, triazoles, thiazole and tetrazole, containing heterocyclic compounds, could display potential antibacterial activity [22,23]; it is also reported [31] that effective antibacterial candidates already screened for their in vitro antibacterial activities are being developed. In particular, it has already been demonstrated that staphylococci, like other gram-positive bacteria, are sensitive to both imidazole [32] and Klebsiella are sensitive to tetrazole-triazole hybrids, demonstrating a promising in vitro antibacterial activity [31].

Notably, single and double biofilms were inhibited by sub-MIC concentrations of VT-1161, and the eradication of mature mono and polymicrobial biofilms was observed at sub-MIC concentrations; this pointed out that VT-1161 could be an adjuvant in the treatment of biofilm-associated fungal/bacterial infections. The MTT cytotoxicity assay showed that VT-1161 did not affect the viability of the PNT1A cell used, indicating its high specificity against *Candida* cells. Conversely, we demonstrated the antiproliferative action of VT-1161 using a LNCaP tumor cell line, suggesting that it could be a potential anticancer drug; however, further cytotoxicity testing (including in vivo studies) will need to be performed. The adherence of *C. albicans, S. aureus* and *K. pneumoniae* to epithelial cells is considered the initial step in some infectious diseases. Our results revealed a reduced adherence of *Candida* cells to PNT1A cells pre-treated with VT-1161, which is more evident for the two bacteria tested. Obviously, in-depth knowledge of the interaction of the host cell with various microorganisms has important implications for understanding the therapeutic strategies to be adopted. Using *C. elegans* as a model, we showed in this study that, under normal conditions, treatment with different concentrations of VT-1161 did not affect worms’ survival. After infection with *C. albicans, S. aureus* and *K. pneumoniae*, VT-1161 treatment effectively inhibited the damage of infection, thus reducing mortality caused by microorganisms’ pathogenic infection and causing higher survival compared to the infected larvae control group four days post- infection. This suggested that VT-1161 might be a valuable antifungal agent against fungal or bacterial infections.

In conclusion, the emergence of multidrug-resistant *Candida* strains has promoted the development of new antibiofilm agents. The present study shows that VT-1161 inhibits and eradicates *C. albicans* biofilm and dual-species biofilms with *S. aureus* and *K. pneumoniae*, with minimal chemical toxicity against *C. elegans* in vivo and mammalian cells in vitro. Further in vivo experiments using *C. elegans* infected by fungal and/or bacterial strains have shown its therapeutic capacity. Other studies are required to confirm the efficacy of VT-1161, and also to gain insights into its mechanisms of action.

## Figures and Tables

**Table 1:** Minimum inhibitory concentration (MIC) of VT-1161, fluconazole (FLC), meropenem (MEM) and vancomycin (VAN) against *C. albicans, K. pneumoniae* and *S. aureus*.

	MIC (µgmL^−1^)			
	VT-1161	FLC	MEM	VAN
*C. albicans*	2.0	16.0	-	-
*K. pneumoniae*	0.5	-	1.0	-
*S. aureus*	0.5	-	-	1.0

## Data Availability

Not applicable.
